# A Mobile Sensing App to Monitor Youth Mental Health: Observational Pilot Study

**DOI:** 10.2196/20638

**Published:** 2021-10-26

**Authors:** Lucy MacLeod, Banuchitra Suruliraj, Dominik Gall, Kitti Bessenyei, Sara Hamm, Isaac Romkey, Alexa Bagnell, Manuel Mattheisen, Viswanath Muthukumaraswamy, Rita Orji, Sandra Meier

**Affiliations:** 1 Department of Psychiatry Dalhousie University Halifax, NS Canada; 2 Faculty of Computer Science Dalhousie University Halifax, NS Canada; 3 Department of Psychology University of Würzburg Würzburg Germany

**Keywords:** mobile sensing, youth, psychiatry, feasibility, mobile phone

## Abstract

**Background:**

Internalizing disorders are the most common psychiatric problems observed among youth in Canada. Sadly, youth with internalizing disorders often avoid seeking clinical help and rarely receive adequate treatment. Current methods of assessing internalizing disorders usually rely on subjective symptom ratings, but internalizing symptoms are frequently underreported, which creates a barrier to the accurate assessment of these symptoms in youth. Therefore, novel assessment tools that use objective data need to be developed to meet the highest standards of reliability, feasibility, scalability, and affordability. Mobile sensing technologies, which unobtrusively record aspects of youth behaviors in their daily lives with the potential to make inferences about their mental health states, offer a possible method of addressing this assessment barrier.

**Objective:**

This study aims to explore whether passively collected smartphone sensor data can be used to predict internalizing symptoms among youth in Canada.

**Methods:**

In this study, the youth participants (N=122) completed self-report assessments of symptoms of anxiety, depression, and attention-deficit hyperactivity disorder. Next, the participants installed an app, which passively collected data about their mobility, screen time, sleep, and social interactions over 2 weeks. Then, we tested whether these passive sensor data could be used to predict internalizing symptoms among these youth participants.

**Results:**

More severe depressive symptoms correlated with more time spent stationary (*r*=0.293; *P*=.003), less mobility (*r*=0.271; *P*=.006), higher light intensity during the night (*r*=0.227; *P*=.02), and fewer outgoing calls (*r*=−0.244; *P*=.03). In contrast, more severe anxiety symptoms correlated with less time spent stationary (*r*=−0.249; *P*=.01) and greater mobility (*r*=0.234; *P*=.02). In addition, youths with higher anxiety scores spent more time on the screen (*r*=0.203; *P*=.049). Finally, adding passively collected smartphone sensor data to the prediction models of internalizing symptoms significantly improved their fit.

**Conclusions:**

Passively collected smartphone sensor data provide a useful way to monitor internalizing symptoms among youth. Although the results replicated findings from adult populations, to ensure clinical utility, they still need to be replicated in larger samples of youth. The work also highlights intervention opportunities via mobile technology to reduce the burden of internalizing symptoms early on.

## Introduction

### Background

Internalizing disorders are the most common psychiatric problems among youth in Canada, with an estimated 6-month prevalence of 11% to 14% and more girls being affected than boys [1]. Over the last 3 decades, a steep increase in the prevalence of internalizing disorders has been observed; unfortunately, this trend is likely to continue [1]. Internalizing disorders typically have an early onset in childhood or adolescence and are characterized by high rates of relapse and chronicity, often resulting in substantial impairment across the lifespan. They are among the top 10 global causes of years lived with disability [2], and severely affect psychosocial development [1], family and social functioning, long-term health outcomes [3], and increased the risk of death by suicide [4]. Unfortunately, only 20% of youth with these disorders receive adequate mental health care [5]. If health professionals cannot predict and efficiently prevent new onset and poor outcomes of internalizing disorders in youth, then this field faces a crisis. Particularly, intervening at critical moments—that is—during mental health crises, including times of risk of suicide, self-harm, psychotic breakdown, substance use relapse, bullying, and interpersonal loss can have a major impact on improving youth mental health.

Current methods of predicting internalizing disorders usually rely on subjective symptom ratings obtained at discrete time points during routine clinical care (eg, clinical monitoring and screening). However, clinical decision-making based on such subjective information is challenging, as changes in symptoms might be subthreshold, context-dependent, or too subtle to be captured using subjective patient ratings. Symptom recall in patients might also not be accurate, potentially biased by current symptom severity [6] and environmental stressors [7]. Moreover, subjective self-reports often fail to reflect symptom variability and context reactivity. Therefore, novel prediction tools need to be developed to meet the highest standards of reliability, feasibility, scalability, and affordability. Given the large proportion of youths who experience mental health crises and do not receive treatment [8], these prediction tools must also encompass those who are not in traditional mental health care.

The recent proliferation of mobile sensing technologies offers health care professionals unprecedented access and insights into youth behavior and mood. Of profound clinical importance is the ability of these technologies to unobtrusively record aspects of youth behaviors in their daily lives with the potential to make inferences about their mental health states [9]. Crucially, these technologies make it possible to obtain detailed moment-by-moment information on youth behaviors and mood while requiring only minimal, if any, active involvement of the users [10]. Smartphones may be especially helpful prediction tools, not only because they afford a wide variety of types of behavioral data that can be automatically recorded by their built-in sensors, but also because they are integral to a youth’s life and nearly 80% of youths own at least 1 smartphone. Thus, smartphones might allow long-term clinical monitoring of internalizing disorders at low cost and high scalability. Collecting high-quality, passive mobile sensing data might further enable the generation of predictive algorithms for solving previously intractable problems and identifying risk states before they manifest as full-blown internalizing disorders.

### Previous Work

The utility of mobile sensing approaches to predict internalizing symptoms has been examined in multiple studies involving adult populations. Several mobile sensor features mirror internalizing symptoms in these studies [11-14]. In particular, GPS data have been shown to be of value in the prediction of internalizing symptoms. For example, the number of different locations visited was found to be negatively associated with social anxiety [11,12] and depression [15-17]. Study participants with social anxiety and depression were also found to avoid public areas and engage in less leisure activities, choosing instead to spend more time at home after school [11,12]. Studies exploring phone use data have reported the number of outgoing calls and messages to negatively correlate with internalizing symptoms [13,15,18]. In addition, ambient light data, an indicator of quality of sleep, were found to be associated with internalizing symptoms [19]. However, to date, mobile sensing studies in the context of youth have been limited [20]. We are only aware of a small pilot study exploring the utility of an Android mobile sensing app among 11 youth patients diagnosed with a major depressive disorder [21]. In that study, the daily number of steps, SMS frequency, and average call duration across a 2-week window were found to be highly correlated with the clinical symptoms of depression. However, in contrast to many studies among adults [17,22], clinical symptoms of depression correlated positively with entropy among youth [21]. This is in line with findings from Jongs et al [22], who also described strong age-related differences in entropy and other GPS-related measures of mobility in a sample aged 18-90 years. Similarly, phone use patterns have been shown to vary between youths and adults. Using a mobile sensing approach, Christensen et al [23] observed that younger age was significantly associated with more screen time. Importantly, screen time has been suggested to be differentially related to internalizing symptoms across age groups [24]. Thus, it is important to explore whether the mobile sensor features found to mirror internalizing symptoms in adults also predict such symptoms in youth.

### Study Aims

Accordingly, this study aims to test the utility of passively collected smartphone sensor data (ie, incoming and outgoing calls, text messages, and GPS data), gathered over 14 days via a mobile sensing app, as the predictors of internalizing symptoms in youth. Studies have shown that internalizing symptoms are common in the general population [25], and the combination of patient and general population samples is meaningful for examining this pathology on a continuum. Therefore, this study aims to collect data from both clinical and nonclinical youth populations. Moreover, the study also uses a mobile sensing app that works on both iOS and Android operating systems, ensuring the generalizability of our results. By including iOS and Android users from clinical and nonclinical settings, we have advanced the inclusivity of current mobile sensing studies in youth [21]. In addition, we hypothesize that mobile sensing data will be associated with internalizing symptoms among youths aged 10-21 years. As outlined, the only study using mobile sensing to predict internalizing symptoms in youth included only 11 participants, thus requiring further evaluation in a larger and more diverse sample. This is especially important as studies have suggested age-related differences in mobile sensing patterns. In contrast to previous studies, we also aim to correct for clinical comorbidities, thus enabling the identification of symptom-specific associations of mobile sensing data. Finally, given the novel nature of these data, we have some exploratory aims, namely, whether the data indicate any nuanced biomarkers that may be worth examining in future iterations of this work.

## Methods

### Overview

Youth participants from clinical and nonclinical populations were recruited for a 14-day observational study, in which a custom app was installed on their personal Android or iOS phone. Self-report measures of anxiety and depression were collected at the beginning of the study. Throughout the duration of the study, the smartphone app passively recorded multiple indices of the youths’ daily life behaviors in an unobtrusive manner. The indices were selected based on findings that demonstrated their potential to make inferences about mental health states [26,27]. These indices included geolocation, sleep, phone use, calls, and messages. A set of features were designed and used to extract higher-level information from these data. Statistical analyses were performed to determine whether a significant relationship existed between participants’ self-reported symptoms of anxiety or depression and these features. The Sciences Research Ethics Board of the Izaak Walton Killam Health Centre in Halifax, (Nova Scotia, Canada) approved this study, which also included the collection of location data.

### Study Procedure

Youth were recruited via posters and social media from the outpatient psychiatry clinic at Izaak Walton Killam Health Centre and the local community in Halifax between February 2020 and July 2020. Youths interested in the study were referred over a link to the lab’s screening page. The study inclusion criteria were as follows: subjects should (1) be between 10 and 21 years old, (2) reside in Canada, (3) be fluent in English, and (4) have an Android or iOS phone that would be in use as the primary mobile phone during the study period (14 days). There were no exclusion criteria for this study. Youth participants who met the inclusion criteria could read a description of the study, which included an informed consent guide. There was no deception nor were the study aims omitted for the participants. All participants provided written informed consent before participation. For youths aged 14 or younger, we required, in addition to their own assent, one of their parents to consent to their study participation. After providing consent, the participants were asked to complete a short web-based survey regarding their clinical symptomatology.

Next, the youth participants were sent instructions to install the app and provided with individualized log-in credentials for using it. Once installed, the study app guided the youths through a short setup, during which they were asked to provide the app with the necessary permissions to access their data, followed by a log-in. Immediately following the setup and log-in, the app began to continuously collect data in the background. No further actions or interactions with the study app were performed until the end of the study, and data were uploaded for 14 days. At this time, the youths received a notification, informing them that the study had ended and directed them to uninstall the app from their phone. A study ID was assigned to each participant, and documents connecting the ID and the participant’s name, demographics, and clinical data were kept separate. The data on the phone were anonymous and only identifiable through the participants’ study ID. To encourage youths to participate in our study, we offered them a monetary reward of Can $20.00 (US $15.82). The REDCap (Research Electronic Data Capture) platform [28] was used for web-based screening, consent, and clinical surveys. REDCap is a secured web-based app that allows researchers to collect, store, and manipulate research data.

### Self-report Assessment

Youth participants completed two self-report measures of internalizing symptoms in digital form at the beginning of the 14-day study. Anxiety symptoms were assessed using the Screen for Child Anxiety-Related Emotional Disorders (SCARED) [29]. SCARED is a self-report instrument for children and their parents, including 41 items that screen for several types of 4 anxiety disorders, namely, generalized anxiety disorder, separation anxiety disorder, panic disorder, and social anxiety disorder [30]. Both the child and parent versions of SCARED in clinical samples demonstrated good internal consistency (α=.74-.93), test-retest reliability (intraclass correlation coefficients 0.70-0.90), and discriminative validity (both between anxiety and other disorders and within anxiety disorders) [29,30]. We administered a 33-item version of SCARED, excluding items related to separation anxiety disorders.

Depression symptoms were assessed using the Center for Epidemiological Studies Depression Scale for Children (CES-DC), which includes 20 items. CES-DC screens for behavioral and cognitive components of depression [31]. The sensitivity of the CES-DC is 85% and can be considered good [32]. In addition, the CES-DC showed results similar to the results of other highly validated instruments [33,34].

To be able to adjust for comorbid externalizing symptomatology, we also required the youths to complete the Adult Attention-Deficit Hyperactivity Disorder Self-Report Scale (ASRS) with adolescent prompts. On the basis of an 18-item scale, the ASRS assesses hyperactivity or impulsivity and inattentiveness symptoms. The ASRS demonstrated a good internal consistency (α=.80) and reliability (α=.79) in adolescent samples [35]. The SCARED, CES-DC, and ASRS instruments asked youth participants to evaluate their symptoms over the past 3 months.

### Smartphone Data Collection

**Figure 1:**
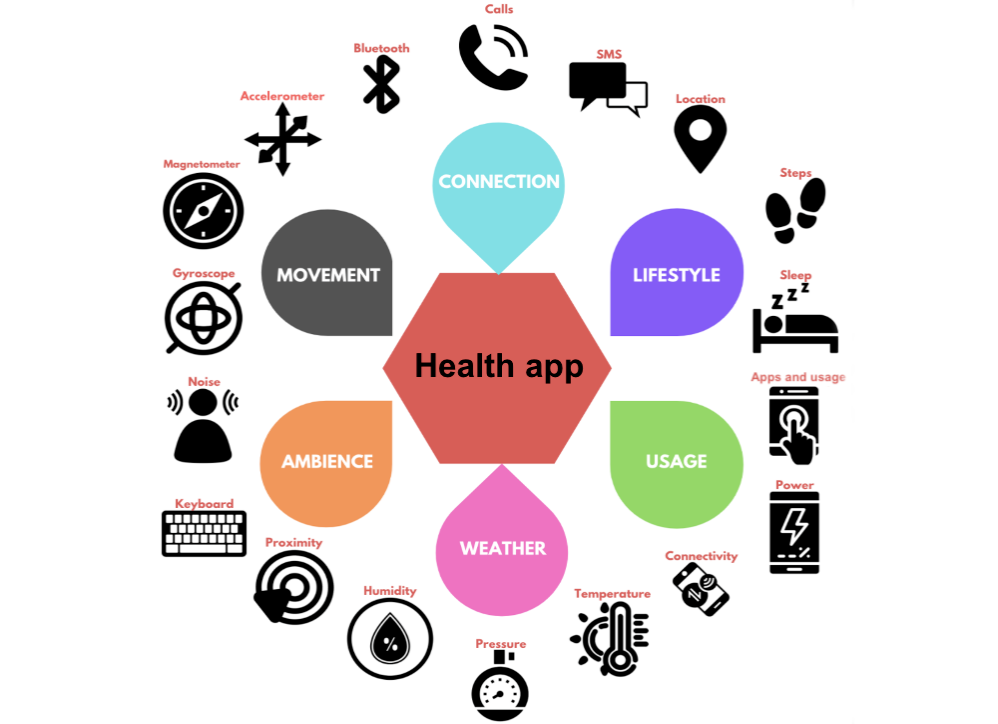
Sensors used to capture behavioral traits.

A mobile sensing app was designed and created to collect all the study data. The PROSIT (Predicting Risk and Outcomes of Social Interactions) tool captures multiple indices of a youth’s daily life behaviors via the naturalistic use of a smartphone (Figure 1). In this study, we used the following features of the PROSIT tool: first, the tool collected GPS data every time the youths moved within a 20-meter radius, preventing the identification of their home address or precise geographical location to preserve privacy. Second, the app logged call events (ie, time and date of the call, duration, and contact of both incoming and outgoing calls), and screen on or off events (ie, time and date). Finally, we sampled ambient light data as an indicator of sleep using the PROSIT tool. Calls and phone use were event-based sensor streams, whereas GPS and ambient light data were sampled as time series. Upon installation, the PROSIT tool continuously ran in the background and collected sensor and use data. The data were first stored locally and then automatically uploaded every hour to a remote server when the phone was in an idle state, and the Wi-Fi was connected. To protect user privacy, all collected sensitive personal data, such as contact details (ie, names and phone numbers), were anonymized during data collection by the app through the built-in cryptographic hash functions, thus guaranteeing that user identity was never recorded either locally or remotely. As the tool is not yet distributed through the official mobile app stores from Apple and Google, a TestFlight-based distribution for iOS and downloadable Android packages for Android was used in this study. The iOS version of the tool was built in the native Swift language using XCode 13 (Apple), whereas the Android version was built using Java and Kotlin languages using Android Studio 4.1 (Google). Importantly, the PROSIT tool also captured steps, notifications, installed apps, app use time, typed text, and music listened to. However, we decided not to include these measures in the current analyses, as these data were slightly differently sampled among Android and iOS users because of certain iOS-specific restrictions. More information on the PROSIT tool is presented in Multimedia Appendix 1 [13,36-50].

### Participant

A total of 161 youth participants downloaded the PROSIT tool into their phone, 6.8% (11/161) withdrew from the study (only 3/161, 1.8% due to privacy concerns), 15.5% (25/161) provided less than 14 days of data, and 1.8% (3/161) did not provide data on sensors relevant to this study. Therefore, this study focused on 75.7% (122/161) of the initial youth participants.

Youth participants were, on average, 18 (SD 2.76) years old and 78.6% (96/122) were female. A total of 5.7% (7/122) participants identified themselves as indigenous, 5.7% (7/122) as black Canadian, 6.5% (8/122) as Asian, and the other 81.9% (100/122) were identified as non-Hispanic Caucasians. As a marker of the youths’ socioeconomic status, we obtained information on maternal education: 4.1% (5/122) of the youths’ mothers had not finished high school, 14.7% (18/122) had finished high school, 23.7% (29/122) had received further education, 54.1% (66/122) had attended university, and 3.2% (4/122) cases lacked this information. A total of 78.6% (96/122) youths were iOS users and 21.3% (26/122) were Android users. In total, 24.5% (30/122) of the youth participants had a lifetime diagnosis of depression, 31.9% (39/122) had a lifetime diagnosis of generalized anxiety disorder, and 9.8% (12/122) had a lifetime diagnosis of social phobia. Regarding the self-report measures of internalizing symptoms, SCARED mean 33.02 (SD 14.79) and range 3-62, the CES-DC mean 32.59 (SD 15.23) and range 1-58. During the study period, 9.8% (12/122) youths were diagnosed with depression, 11.4% (14/122) were diagnosed with generalized anxiety, and 4.9% (6/122) were diagnosed with social phobia. Finally, we excluded two participants from the call analyses and one participant from the GPS analyses, as they were extreme outliers.

### Feature Extraction

Raw unobtrusive mobile sensing data with respect to mobility, social interactions, phone use, and sleep were aggregated into daily summaries. As outlined above, we logged each GPS data point when the displacement from the last point was larger than 20 m. From these data, we extracted the number of locations visited per hour for at least 2 minutes. The distance between stationary states is defined by the Haversine distance function, which calculates the distance between a pair of coordinates denoted by latitude and longitude. We extracted the total distance traveled (times traveled a day), normalized entropy, and time spent at one location (ie, stationary at work or at home), 2-9 locations (ie, moving), and over 10 locations (ie, transportation) per hour for each participant on a daily basis. Normalized entropy was defined as

Normalized Entropy = −∑^N^_i_ p_i_log p_i_(2) / log N **(1)**

where each i=1, 2,..., N represented a location cluster (in a 150-m radius), N denoted the total number of location clusters, and p_i_ was the percentage of time the participant spent at the location cluster i. High entropy indicated that the participant spent time more uniformly across different location clusters, whereas lower entropy indicated a greater inequality in the time spent across the clusters. Unlike entropy, the normalized entropy is invariant to the number of clusters; thus, it depends solely on the distribution of the visited location clusters. The value of normalized entropy ranges from 0 to 1, where 0 indicates that all location data points belong to the same cluster, and 1 implies that they are uniformly distributed across all clusters. For each phone call, we logged the timestamp, type (incoming, outgoing, and missing), and duration. For each user, we computed the total number of phone calls (connected, incoming, and outgoing) and total call duration on a daily basis. The raw mobile phone screen on or off events were transformed into two features: (1) the total number of times the screen was turned on per day and (2) the total amount of screen time per day (calculated as the difference between the number of screen on or off events). From the ambient light data, we logged changes in light intensity and calculated the time spent at each light intensity level. Next, we computed the total average light intensity, time spent in the lowest and highest light intensity ranges during nighttime per day (11 PM-7 AM), which was used as an indicator of sleep quality.

### Statistical Analysis

After completing feature extraction and exclusion of extreme outliers, we performed the Spearman correlation analysis of sensor data over 14 days with SCARED and CES-DC scores of youth participants at a dimensional level. All analyses were adjusted for age, sex, and maternal education as the indicators of socioeconomic status, comorbidities, and the smartphone operating system. In addition, we included the month of assessment to account for potential impact of the pandemic on the results. We also fitted linear regressors by comparing models with and without passively collected smartphone sensor data as predictors. Finally, we assessed the discriminant validity of the predicted internalizing symptoms; for example, we ran correlations between the SCARED scores based on smartphone biomarkers and CES-DC and ASRS scores.

## Results

### Mobile Sensing Data Characteristics

The mean and SD of mobile sensing features across youth participants are presented in Table 1. To predict the assessment of internalizing symptoms (SCARED and CES-DC), we used averages across individuals as predictor variables.

**Table 1:** Descriptive statistics of mobile sensor data and features.

Feature			Values, mean (SD)
**Mobility**			
	Time sedentary	18.82 (3.60)	
	Time moving	4.02 (3.56)	
	Distance traveled	6.01 (3.56)	
	Entropy	0.48 (0.20)	
**Social interaction**			
	Call duration	15.22 (13.63)	
	Incoming calls	1.81 (0.94)	
	Outgoing calls	2.11 (1.20)	
	Connected calls	2.30 (1.25)	
**Phone usage**			
	Screen use time	249.58 (109.28)	
	Screen use unlocks	53.66 (38.71)	
**Sleep**			
	Ambient light intensity	304,883 (95,828)	
**Clinical symptoms**			
	SCARED^a^	33.02 (14.79)	
	CES-DC^b^	32.59 (15.23)	

^a^SCARED: Screen for Child Anxiety-Related Emotional Disorders.

^b^CES-DC: Center for Epidemiological Studies Depression Scale for Children.

### Relationship Between Smartphone Data and Psychometric Scores

We used the pairwise Spearman coefficient as an indicator of the correlations between mobile sensing data and clinical self-report measures of internalizing symptoms (SCARED, CES-DC). The mobile sensing data were represented by mobility, social interaction, phone use, and sleep-related features extracted using methods described in Feature Extraction and Statistical Analysis of the Methods section. The analysis and correlations are shown in Tables 2 and 3.

Table 2 shows the correlations between mobility and internalizing symptom scores, where mobility is captured using GPS data. From the table, we observe that youths with more severe depression and higher CES-DC scores spent more time stationary (*r*=0.293; *P*=.003) and were significantly less mobile (*r*=−0.271; *P*=.006). In contrast, less time spent stationary (*r*=−0.249; *P*=.01) and higher mobility (*r*=0.234; *P*=.02) were associated with more anxiety symptoms as measured by SCARED. No correlations between normalized entropy values and internalizing symptoms were observed.

The correlations of social interactions, phone use, and sleep-related features with internalizing symptom scores are shown in Table 3. The results indicate that higher depression scores are significantly correlated with lower social interaction levels, such as fewer outgoing phone calls (*r*=−0.223; *P*=.03). Higher anxiety scores also correlated with fewer outgoing phone calls (*r*=−0.293; *P*=.003); however, this correlation became insignificant after adjusting for comorbid depression (*r*=−0.069; *P*=.50). In addition, higher ambient light intensity during the night, which is a proxy for poorer sleep quality, was correlated with higher depression scores (*r*=0.227; *P*=.02), but not anxiety scores (*r*=−0.15; *P*=.12). Conversely, higher smartphone screen use was only correlated with higher anxiety scores (*r*=0.203; *P*=.049).

**Table 2:** Spearman correlation coefficients between internalizing symptoms and mobility features.

Feature	Anxiety			Depression	
	Correlation coefficient (*r*)	*P* value	Correlation coefficient (*r*)		*P* value
Distance travelled	0.230	.02	−0.271		.006
Time moving	−0.248	.01	0.293		.003
Time sedentary	0.234	.02	−0.266		.007
Entropy	0.076	.46	−0.042		.69

**Table 3:** Spearman correlation coefficients between internalizing symptoms and social interactions, phone use, sleep-related features.

Feature	Anxiety			Depression	
	Correlation coefficient (*r*)	*P* value	Correlation coefficient (*r*)		*P* value
Call duration	0.024	.81	−0.035		.73
Incoming calls	0	.99	−0.072		.48
Outgoing calls	−0.069	.50	−0.223		.03
Connected calls	−0.106	.31	−0.110		.29
Ambient light intensity	−0.136	.12	0.227		.02
Screen use	0.203	.049	−0.113		.28

Thus, internalizing symptoms assessed by clinical instruments were significantly correlated with mobility levels and social interactions captured by passive smartphone sensor data. We also observed significant correlations between other daily living contexts (ie, ambient light intensity and smartphone screen use) and internalizing symptoms.

The inclusion of mobility, social interactions, phone use, and sleep-related features significantly improved the fit of models predicting more severe depression and higher CES-DC scores (*F*_17,62_=2.036; *P*=.04), as well as more severe anxiety and higher SCARED scores (*F*_17,62=_2.256; *P*=.02). Finally, predicted SCARED scores from smartphone biomarkers correlated with higher depression scores (*r*=0.258; *P*=.01), but not ASRS scores (*r*=0.062; *P*=.55). Consistently, predicted CES-DC scores correlated with higher anxiety scores (*r*=0.258; *P*=.01), but not ASRS scores (*r*=0.020; *P*=.85).

## Discussion

### Principal Findings

Our results support the hypothesis that passively collected smartphone sensor data can provide sufficient information for successfully predicting internalizing symptoms among youth. The proposed approach worked well for youths who were usually heavy smartphone users. Remarkably, we were able to include youths as young as 10 years of age in our completely remote study design, demonstrating the feasibility of mobile sensing among youth. The data also underlined high acceptability; even youths with severe clinical symptomology reported only minimal issues in the use of the app. Dropouts due to privacy concerns can be considered low (3/161, 1.9%).

The study indicated that there were significant correlations between internalizing symptoms and smartphone biomarkers. Lower levels of mobility, fewer social interactions, and higher ambient light intensity during the night, used as a proxy for poorer sleep quality, were correlated with higher depression symptoms. Higher levels of mobility, fewer social interactions, and higher smartphone screen use were correlated with higher anxiety symptoms among youth. The study also showed that by adding passively collected smartphone sensor data to prediction models, we were able to achieve better model fits. Finally, the results indicate the potential of smartphone biomarkers to have a discriminative nature by predicting other internalizing symptoms but not adult attention-deficit hyperactivity disorder symptoms.

Here, we expand on the only previous study among youth showing that light intensity and smartphone screen use in addition to mobility and social interactions might be valuable features for predicting internalizing symptoms among youth [21]. In addition, we confirmed the potential discriminative validity of smartphone biomarkers observed among adults in a youth sample [51]. Although our results on the number of outgoing calls negatively correlating with internalizing symptoms are in line with previous studies in adults [13,15,18], differences in mobility patterns of youths with high anxiety symptoms as compared with high depression symptoms have not been described thus far [11,14,21]. However, these findings have to be interpreted considering the recent pandemic and the behavioral changes accompanying it [52]. It is easy to argue that the time spent outside during a pandemic might increase anxiety symptoms.

### Limitations and Future Directions

This study has some limitations that provide directions for future research. First, the conclusions must be considered preliminary, given the relatively small sample size of 122 youths and require further replication. Larger samples will also allow for more power to assess potentially moderating effects (eg, sex, trauma exposure), especially as the cost-effectiveness of mobile sensing by using the patients’ own smartphones is fostering the scalability of the approach. However, in contrast to many previous studies [11], we were able to work with a relatively heterogeneous real-world sample of youths, including patients with severe symptomatology, which may reduce the generalizability of our findings. Second, through the use of passively collected smartphone sensor data, we may miss relevant information on social interactions; for example, it can be difficult to infer the quality of the social contact based on the metadata of call logs. Third, our study focused on identifying between-person differences; the identified predictors might not be of value in predicting within-person variability of internalizing symptoms. Fourth, it is not possible to make any claims regarding causation between mobility, screen time, sleep, social interactions, and internalizing symptoms. It cannot be determined if fewer social interactions lead to more internalizing symptoms or vice versa. Fifth, our study was conducted during the recent pandemic that led to a multitude of behavioral changes [52,53]; thus, the results might not be generalizable to a postpandemic situation. However, our study findings were largely consistent with those of previous studies conducted in adult populations, supporting the reliability of mobile sensing results.

The next steps should focus on exploring how youth patients, their parents, and clinicians would prefer to be notified about the youths’ passively collected smartphone sensor data, what inferences can be drawn about youths’ mental health states from these data, and what actions they would find useful and acceptable in response to these data. For example, early warning signs can be identified from smartphone biomarkers, triggering early interventions. In addition, the enthusiasm for exciting new possibilities associated with passively collected smartphone sensor data must be carefully evaluated regarding the challenges of privacy and data security.

### Conclusions

In sum, our study expands recent efforts to use passively collected smartphone sensor data for predicting internalizing symptoms among youth. Such accurate behavioral assessments might be especially important for youths with internalizing symptoms, given that the occurrence of these symptoms is more frequent than is typically reported. However, this method of harnessing naturally occurring behavioral data is certainly relevant for identifying and better understanding a range of maladaptive thoughts and behaviors that underlie psychiatric conditions more broadly. Finally, it is equally important to be sensitive and treat passively collected smartphone sensor data as sensitive health information and ensure adequate privacy and security controls are in place before wider use.

## Appendix

Multimedia Appendix 1 The PROSIT (Predicting Risk and Outcomes of Social Interactions) app.

## Abbreviations

ASRS: Adult Attention-Deficit Hyperactivity Disorder Self-Report Scale
CES-DC: Center for Epidemiological Studies Depression Scale for Children
PROSIT: Predicting Risk and Outcomes of Social Interactions
REDCap: Research Electronic Data Capture
SCARED: Screen for Child Anxiety-Related Emotional Disorders
