# Supplementary material for: A Mobile Sensing App to Monitor Youth Mental Health: Observational Pilot Study
Source: JMIR Mhealth Uhealth. 2021 Oct 26;9(10):e20638. doi: 10.2196/20638 (PMC8579216; doi:10.2196/20638)
Supplement: Multimedia Appendix 1 [file mhealth_v9i10e20638_app1.docx]

**Multimedia Appendix 1.** The PROSIT (Predicting Risk and Outcomes of Social Interactions) app.


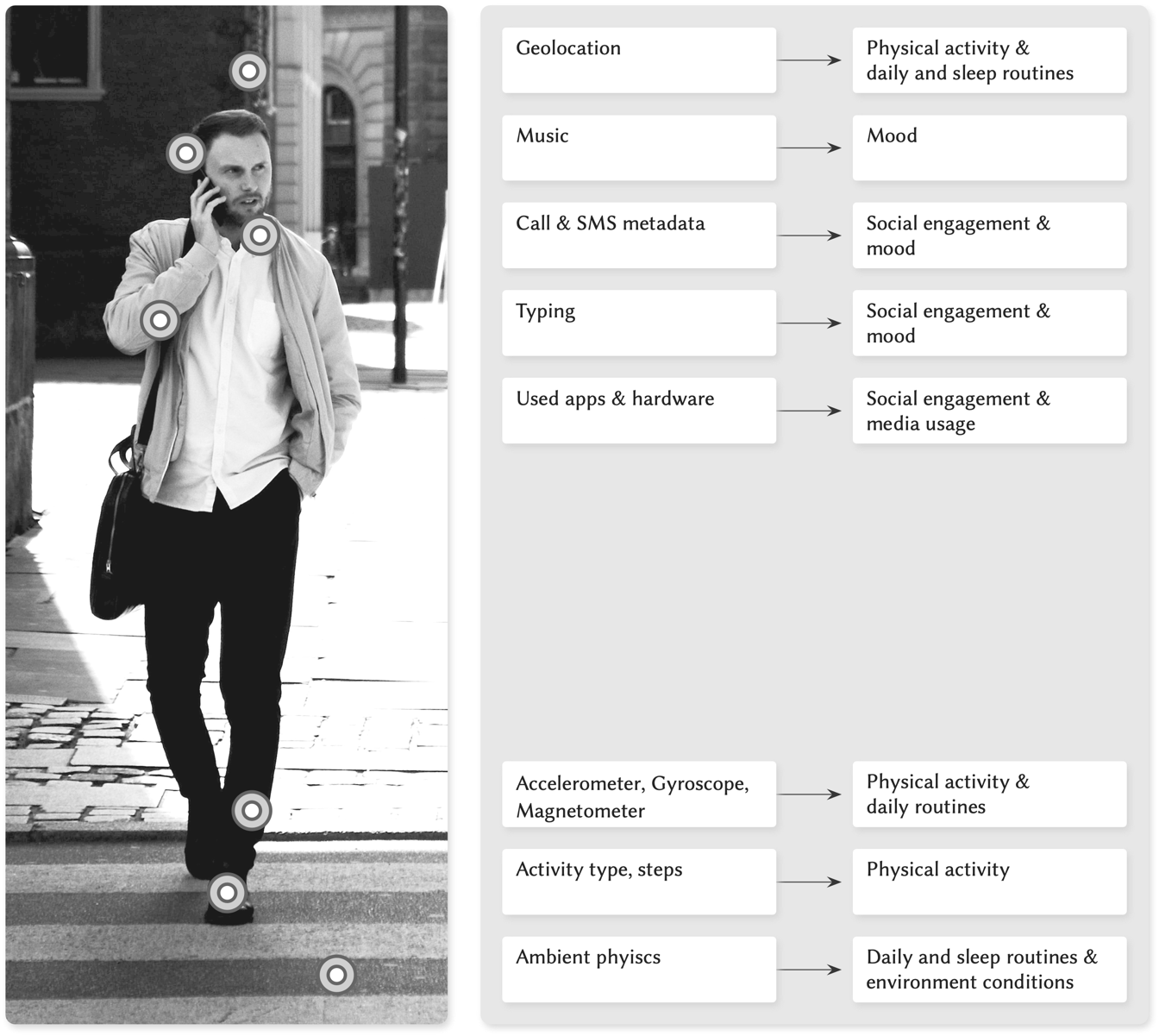


*Figure S1:* An overview of how mobile sensing data might relate to clinical symptomatology.

Physical activity has a rich history of positive outcomes for mental health. A number of studies suggested that physical activity reduces the risk for mental disorder through physiological pathways. For example, physical activity may activate endorphin secretion, which reduces pain and produces a euphoric sensation [36]. Research also indicates that physical activity benefits brain development [37]. Another theory concerning why physical activity is helpful in promoting mental health suggests that exercise may alleviate symptoms of emotional problems rather than the emotional problem itself. For example, some depressive symptoms are somatic in nature (e.g., disturbed sleep, general fatigue, diminished appetite); therefore, improvements in these types of indicators of depression might reflect the general benefits of physical activity rather than the physical activity having any direct impact on the depression itself. Geolocation overlaps with physical activity in part, but it can also provide important insight into the quality of the environments in which youth are spending their time. Environmental factors indexed by Global Positioning System (GPS) data, such as living in a city and being exposed to green areas, were found to have consequences for social stress processing and long-term mental health outcomes [38, 39]. A summary of these indices is displayed in Figure S1.

Sleep is another powerful predictor of mental health state. Sleep is vital for youth’s brain function and behavior [40] because of its involvement in brain development, learning, memory, and emotion regulation. Sleep and daily rhythm disruptions are seen in a number of mental disorders, including depression, bipolar disorder, attention-deficit/hyperactivity disorder, schizophrenia and anxiety [41]. Altered sleep is not only a symptom of these disorders, but also plays an important role in triggering and maintaining some conditions. Thus, sleep loss or altered daily rhythm timing can trigger mania in bipolar patients [42], persistent sleep problems increase the risk of relapse in depression, and targeted treatment of insomnia symptoms improves depression symptoms and reduces the risk of relapse in patients [43]. Evidence suggests that phone use duration may affect mental disorders via sleep disturbance [44]. For instance, spending a long time on the phone might lead to reduced sleep duration, whilst incoming alerts in the night and fear of missing out on new content could cause sleep disruption [45, 46]. Screen exposure before bedtime and the consequent adverse impact of this on melatonin production and the circadian rhythm are also possible mechanisms [47]. Finally, sleep quality and quantity could also be affected by levels of stress/worry from cyberbullying experiences.

However, phone usage also deserves study in its own right. A study exploring phone usage data reported the number of outgoing calls and messages to negatively correlate with symptoms of depression and anxiety [13]. Typed text such as social media posts can configure valuable predictors of mental health states. In example, based on natural language processing of Facebook statuses posted by 683 patients visiting a large urban academic emergency department, Eichstaedt at al. were able to predict diagnoses of depression with fair accuracy [48]. In addition, music choices provide great insights in mental health states, as recent findings suggest that listeners choose music to satisfy emotional needs, especially during periods of negative mood [49]. Finally, several aspects of acoustic vocal quality provide robust indicators of mental health states. These aspects include speech rate, vocal prosody, vowel space, and other machine learning-derived features [50].

The PROSIT tool includes the following 7 features. First, the tool constantly collects inertial measurement unit data making use of the smartphone’s built-in accelerometer, gyroscope, and magnetometer sensors. This enables the fine-grained estimation of physical activity, over and above what can be gleaned from Google Fit and Apple Health Kit data. Second, the tool is configured to collect GPS data for every time youth move within a 20-meter radius preventing identification of youth’s home address or precise geographical location to preserve privacy. Paired with the inertial measurement unit data, the ability to sample ambient light and noise via the smartphone’s sensors further enhances the PROSIT tool’s measurement of the third feature, sleep. Fourth, the tool permits the automatic collection of 7 indices of phone use: SMS frequency, call frequency, notifications, screen-on time, installed apps, and app use time. Fifth, meta-data typed text is recorded, such as text length, number of letters, numbers, and emoticons used. The sixth feature monitors the notification center to capture what music youth listen to across various music apps. Lastly, acoustic voice features are sampled through youth’s audio diaries.

In addition to these multiple indices of a youth’s daily life behavior the PROSIT tool records weather, battery life, and device information. The tool also facilitates integration with wearable technology to collect raw data from wearable devices (e.g., wrist wearables that measure actigraphy and heart rate). This integration of wearable technology improves efficiency of data collection by eliminating the need of signing into each individual wearable account to download these data. Capturing raw wearable data yields physiological variables that are unobscured by preprocessing. Finally, the tool sends questionnaires to user’s smartphone. The questionnaire definitions and their regimen are defined by simple JSON configuration files which are web-served and therefore remotely configurable. New questionnaire configuration files can be easily created either manually or authored as REDCap data dictionaries and parsed via a simple script. Figure S2 shows a list of all variables that can be recorded by the PROSIT tool.

Every hour, all mobile-sensed data is transferred to the database on a secure server through the RESTful API if the PROSIT tool is on the Internet, otherwise the data is locally stored until the device gets an Internet connection. The tool is not yet distributed through the official mobile app stores from Apple and Google, currently a TestFlight-based distribution for iOS and downloadable Android packages for Android is used. Presently, the PROSIT is available in English/German; however, we aim to translate it to other languages such as French.


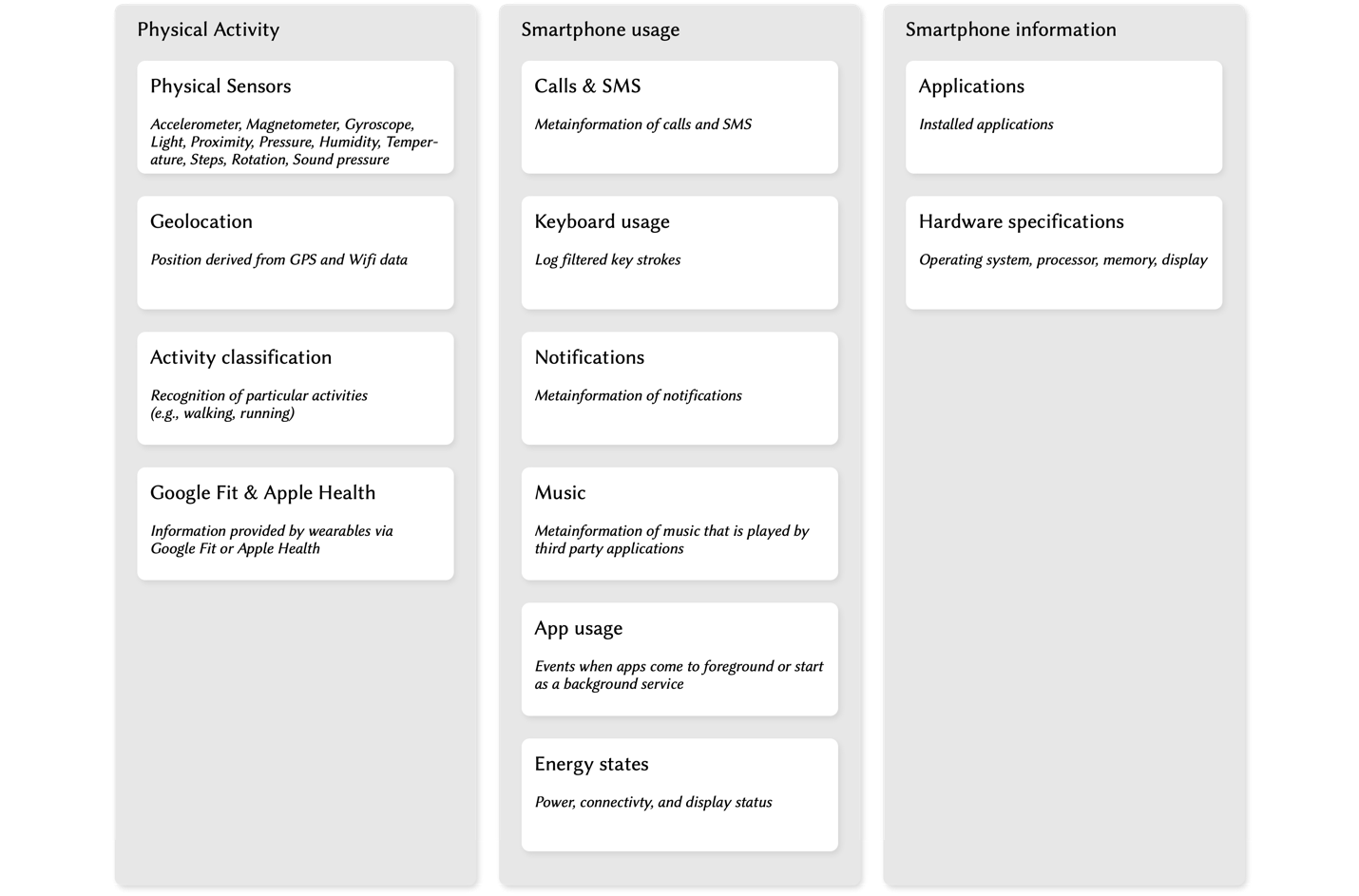


Figure S2: Overview of collected data. The PROSIT Android and iOS applications collect data that reflect the physical activity, sleep, and the smartphone usage of the user, as well as more stable smartphone specifications.

**PROSIT Design**

The two main considerations guiding the design of the PROSIT tool were (1) privacy and encryption, and (2) phone use impact. To detect risk states before they become mental health crises the PROSIT tool collects a massive amount of personal data, so it is imperative that these data remain secure and cannot be used to identify youth. To ensure data privacy, we have implemented a detailed security protocol to deidentify and encrypt all mobile-sensed data. First the identity of youth is anonymized. Neither the name, the phone number, nor the phone ID is stored. Instead, the PROSIT tool assigns youth a 9 character long unique identifier, called “Study ID”, to each user. Also, all sensitive information collected by the PROSIT tool is stored inside a private database, which is not accessible by any third-party software. Obviously, these basic first steps do not safeguard the actual content of the data.

Thus, state-of-the-art encryption is implemented in the data security pipeline. After the sensors generate data, the data are immediately encrypted by the PROSIT tool using 128-bit Advanced Encryption Standard (AES) encryption, a government standard endorsed by the US National Institute of Standards and Technology. Upon encryption, the unencrypted data are immediately deleted. Moreover, all phone numbers and names of persons are irrevocably anonymized by a cryptographic hash function. The big benefit of cryptographic hashing is that it is still possible to inspect social networks of youth, since the same contact on youth’s phones will result in the same hash value. Also, there is an additional security protocol in place to handle all typed text, all typed text can be fully read by the PROSIT tool. The content of all typed text will not leave the phone but will be analyzed directly on the device. Specifically, the tool computes the text length, number of letters, numbers, and emoticons used. During data transfer from mobile phone to server, the data is encrypted using RSA/ECB/OAEPWithSHA-1AndMGF1Padding encryption algorithm. Finally, after the successful data upload to the secure server at our clinic, the PROSIT tool deletes all mobile-sensed data from the phone’s memory.

The user interface of PROSIT is designed using XCode for iOS and Android Studio for Android mobile phones, following the interactive design process and refinement, and in line with the Human Computer Interaction guidelines. Figure S3 shows the screens of the PROSIT tool.


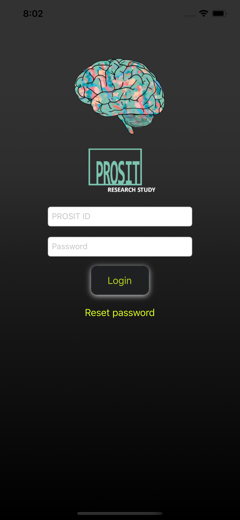

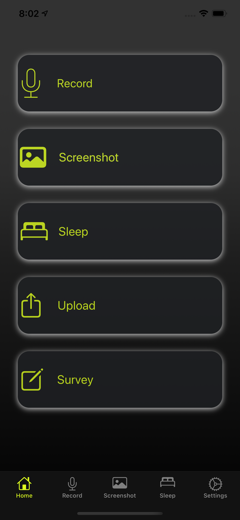

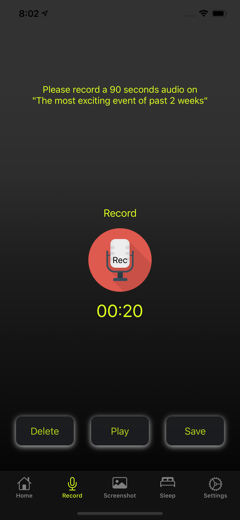


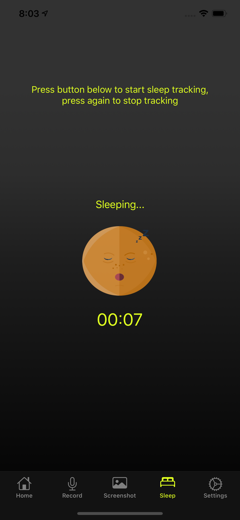

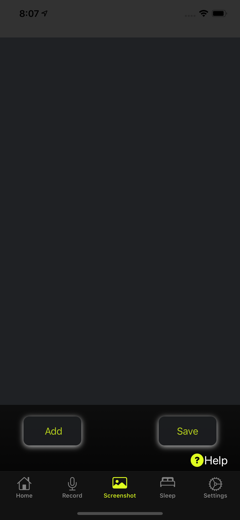

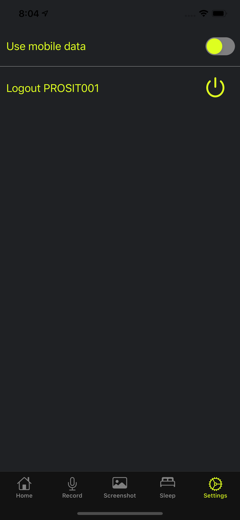


*Figure S4:* PROSIT mobile application screens (for details see supplement).

The PROSIT backend software is installed on the secure server at our clinic ensures that the data is only send to this secure server, protected by Firewall. The PROSIT backend consists of a set of Docker services. They manage the authentication of users of the PROSIT tool, process data upload, and provide a dashboard to manage users and to access data. A reverse proxy Nginx server forwards the incoming requests, ensures secure HTTPS connection, and mitigates Distributed Denial-of-Service attacks. A web server then processes incoming requests. We developed the webserver with the Meteor web framework. The web server provides a REST API for mobile clients. The API provides endpoints to request authentication and to upload data. We follow the JSON Web Token standard to authenticate mobile clients and permit data upload. The web server furthermore provides a dashboard. The dashboard is a browser-based user interface for the administration of users and the access of collected data, also the dashboard is password protected. A MongoDB database stores all textual data and a docker volume stores uploaded image and audio files. The backend has its own access permission system ensuring no third parties have access to the stored data. Both, the PROSIT tool and backend are designed to use SSL for data transmission. SSL encrypts data when it is sent over the Internet and it is the de facto standard for high level of data security. Figure S4 illustrates the architecture of the PROSIT backend.

*
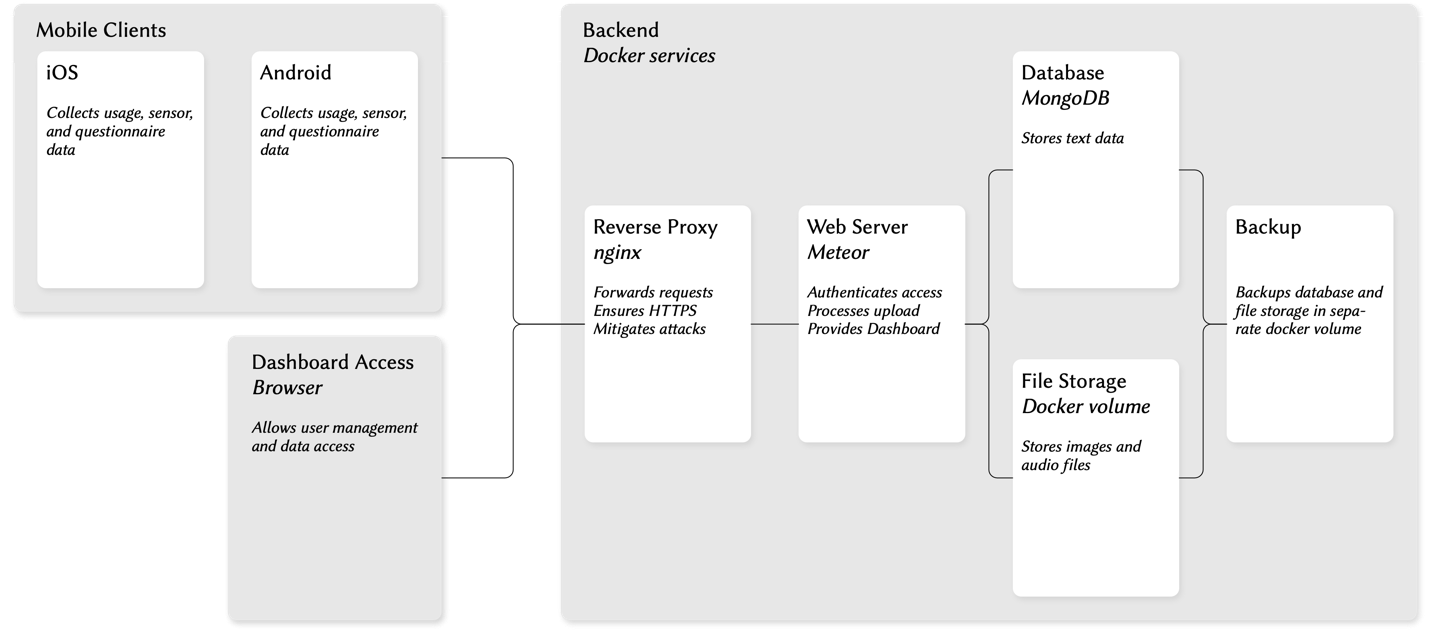
*

Figure S4: The architecture of the PROSIT backend. The backend consists of Docker services that manage user authentication, upload of collected data, and access to the dashboard.

Another important step in designing the PROSIT tool was made towards maximizing acceptability

of the PROSIT tool by prioritizing phone use impact. The PROSIT tool is constantly running in the background of youth’s phone. To limit the impact of the PROSIT tool on the youth’s daily phone usage experience, the tool has been configured as lightweight as possible. First, the tool consumes around 30 MB of RAM. Second, the use of smartphone’s built-in sensors, especially GPS, can have a large impact on the battery life of a phone, as they draw relatively large amounts of power. To combat this, data uploads only happen when the device is connected to the Internet. Current testing on a range of smartphones indicated that the tool consumes approximately 20% of the battery over a 24-hour period. Moreover, installation of the PROSIT tool causes minimal change in the user interface. Once installation is completed, the only difference youth will notice is the custom keyboard in the iOS version of the tool.

**References**

36. Paluska SA, Schwenk TL. Physical activity and mental health: current concepts. Sports Med. 2000 Mar;29(3):167-80. PMID: 10739267. doi: 10.2165/00007256-200029030-00003.

37. Gomes da Silva S, Arida RM. Physical activity and brain development. Expert Rev Neurother. 2015;15(9):1041-51. PMID: 26289488. doi: 10.1586/14737175.2015.1077115.

38. Lederbogen F, Kirsch P, Haddad L, Streit F, Tost H, Schuch P, et al. City living and urban upbringing affect neural social stress processing in humans. Nature. 2011 Jun 22;474(7352):498-501. PMID: 21697947. doi: 10.1038/nature10190.

39. Tost H, Reichert M, Braun U, Reinhard I, Peters R, Lautenbach S, et al. Neural correlates of individual differences in affective benefit of real-life urban green space exposure. Nat Neurosci. 2019 Sep;22(9):1389-93. PMID: 31358990. doi: 10.1038/s41593-019-0451-y.

40. Tarokh L, Saletin JM, Carskadon MA. Sleep in adolescence: Physiology, cognition and mental health. Neurosci Biobehav Rev. 2016 Nov;70:182-8. PMID: 27531236. doi: 10.1016/j.neubiorev.2016.08.008.

41. Walker MP, Harvey AG. Obligate symbiosis: sleep and affect. Sleep Med Rev. 2010 Aug;14(4):215-7. PMID: 20427211. doi: 10.1016/j.smrv.2010.02.003.

42. Wehr TA. Sleep-loss as a possible mediator of diverse causes of mania. Br J Psychiatry. 1991 Oct;159:576-8. PMID: 1751874. doi: 10.1192/bjp.159.4.576.

43. Cheng P, Kalmbach DA, Tallent G, Joseph CL, Espie CA, Drake CL. Depression prevention via digital cognitive behavioral therapy for insomnia: a randomized controlled trial. Sleep. 2019 Oct 9;42(10). PMID: 31535688. doi: 10.1093/sleep/zsz150.

44. Lemola S, Perkinson-Gloor N, Brand S, Dewald-Kaufmann JF, Grob A. Adolescents' electronic media use at night, sleep disturbance, and depressive symptoms in the smartphone age. J Youth Adolesc. 2015 Feb;44(2):405-18. PMID: 25204836. doi: 10.1007/s10964-014-0176-x.

45. Woods HC, Scott H. #Sleepyteens: Social media use in adolescence is associated with poor sleep quality, anxiety, depression and low self-esteem. J Adolesc. 2016 Aug;51:41-9. PMID: 27294324. doi: 10.1016/j.adolescence.2016.05.008.

46. Godsell S, White J. Adolescent perceptions of sleep and influences on sleep behaviour: A qualitative study. J Adolesc. 2019 Jun;73:18-25. PMID: 30953841. doi: 10.1016/j.adolescence.2019.03.010.

47. Touitou Y, Touitou D, Reinberg A. Disruption of adolescents' circadian clock: The vicious circle of media use, exposure to light at night, sleep loss and risk behaviors. J Physiol Paris. 2016 Nov;110(4 Pt B):467-79. PMID: 28487255. doi: 10.1016/j.jphysparis.2017.05.001.

13. Place S, Blanch-Hartigan D, Rubin C, Gorrostieta C, Mead C, Kane J, et al. Behavioral Indicators on a Mobile Sensing Platform Predict Clinically Validated Psychiatric Symptoms of Mood and Anxiety Disorders. J Med Internet Res. 2017 Mar 16;19(3):e75. PMID: 28302595. doi: 10.2196/jmir.6678.

48. Eichstaedt JC, Smith RJ, Merchant RM, Ungar LH, Crutchley P, Preotiuc-Pietro D, et al. Facebook language predicts depression in medical records. Proc Natl Acad Sci U S A. 2018 Oct 30;115(44):11203-8. PMID: 30322910. doi: 10.1073/pnas.1802331115.

49. Noel VA, Acquilano SC, Carpenter-Song E, Drake RE. Use of Mobile and Computer Devices to Support Recovery in People With Serious Mental Illness: Survey Study. JMIR Ment Health. 2019 Feb 20;6(2):e12255. PMID: 30785401. doi: 10.2196/12255.

50. Low LS, Maddage NC, Lech M, Sheeber LB, Allen NB. Detection of clinical depression in adolescents' speech during family interactions. IEEE Trans Biomed Eng. 2011 Mar;58(3):574-86. PMID: 21075715. doi: 10.1109/TBME.2010.2091640.
